# Binocular Summation Revisited: Beyond √2

**DOI:** 10.1037/bul0000163

**Published:** 2018-08-13

**Authors:** Daniel H. Baker, Freya A. Lygo, Tim S. Meese, Mark A. Georgeson

**Affiliations:** 1 Department of Psychology, University of York; 2 School of Life and Health Sciences, Aston University

**Keywords:** binocular summation, meta-analysis, psychophysics, contrast, spatiotemporal frequency

## Abstract

Our ability to detect faint images is better with two eyes than with one, but how great is this improvement? A meta-analysis of 65 studies published across more than 5 decades shows definitively that psychophysical binocular summation (the ratio of binocular to monocular contrast sensitivity) is significantly greater than the canonical value of √2. Several methodological factors were also found to affect summation estimates. Binocular summation was significantly affected by both the spatial and temporal frequency of the stimulus, and stimulus speed (the ratio of temporal to spatial frequency) systematically predicts summation levels, with slow speeds (high spatial and low temporal frequencies) producing the strongest summation. We furthermore show that empirical summation estimates are affected by the ratio of monocular sensitivities, which varies across individuals, and is abnormal in visual disorders such as amblyopia. A simple modeling framework is presented to interpret the results of summation experiments. In combination with the empirical results, this model suggests that there is no single value for binocular summation, but instead that summation ratios depend on methodological factors that influence the strength of a nonlinearity occurring early in the visual pathway, before binocular combination of signals. Best practice methodological guidelines are proposed for obtaining accurate estimates of neural summation in future studies, including those involving patient groups with impaired binocular vision.

## Public Significance Statement

This meta-analysis demonstrates conclusively that vision with two eyes is better than with one, to a greater extent than is generally believed. This provides benchmarks for clinical studies investigating disorders of binocular vision such as amblyopia. We also demonstrate the effect of methodological factors in measuring binocular improvements, and provide best practice guidelines for future studies.

The human visual system pools information across the two eyes to create a single stable representation of the world. At low contrasts near the limit of detectability, sensitivity to variations in luminance is improved by presenting a stimulus to both eyes (binocularly) rather than to only one eye (monocularly). This improvement in sensitivity is known as binocular summation, and has been measured in numerous studies over the past 50 years as an important index of binocular function. Early work (Campbell & Green, 1965) reported that the mean sensitivity improvement was a factor of √2, meaning that, on average, a monocularly presented stimulus requires a contrast 1.4 times higher than the same stimulus presented binocularly in order to be equally detectable. This is consistent with a squaring nonlinearity operating before the two monocular signals are summed physiologically in the cortex (Legge, 1984b). However, more recent work (e.g., Meese, Georgeson, & Baker, 2006) has reported substantially greater improvements, up to a factor of around 1.8, implying a weaker nonlinearity.

Determining the “true” level of binocular summation has been challenging, in part because different studies use a diverse range of stimulus parameters, psychophysical techniques, and analysis methods. In addition, most studies test relatively few observers (median *N* = 5 in the studies we discuss here), meaning that individual differences in binocular vision could have a strong influence on summation estimates. Here, we aim to determine the methodological factors that govern the empirical measurement of binocular improvement. We do this by conducting a meta-analysis of 65 published studies reporting binocular summation of contrast, and confirming these findings with two further data sets that measure binocular summation as a function of spatiotemporal frequency, and individual differences in sensitivity between the eyes. In order to consider these results within a common framework, we first define a minimal model of binocular signal combination at threshold.

## A Canonical Model of Summation at Threshold

We assume that detection decisions are determined by the response of a binocular mechanism, that takes two monocular inputs and sums them together:

resp= L + R 1

where L and R are the contrasts of stimuli presented to the left and right eyes respectively, and performance-limiting late additive noise is approximated by defining threshold at a fixed (but arbitrary) response level (e.g., resp = 1). This linear model predicts that binocular sensitivity (resp_BIN_) is twice that of monocular sensitivity (resp_MON_) because (trivially) 1 + 1 = 2 + 0; when the stimulus is presented to both eyes it requires half the contrast to produce the same response as when it is presented to only one eye. A more general form of the model is given by:

$$\text{resp}={\text{L}}^{m}+{\text{R}}^{m}$$ Figure 2

where the exponent *m* governs the level of summation, for which the summation ratio can be derived precisely as 2^1/*m*^ (Baker, Wallis, Georgeson, & Meese, 2012). When *m* = 1, summation is linear (as in Equation 1), because 2^1/1^ = 2. When *m* = 2, summation is reduced because 2^1/2^ = √2. Subsequent nonlinearities (after the monocular signals are summed) do not affect the level of summation. Obtaining an accurate empirical estimate of binocular summation is therefore informative regarding nonlinearities early in the visual pathway, before information is combined across the eyes. With this aim in mind we conducted a meta-analysis to aggregate summation ratios across more than five decades of published work, for a total sample size of *N* = 716 observers.

## Meta-Analysis

### Meta-Analysis: Method

We collected published studies reporting psychophysical binocular summation ratios (BSR) for luminance-defined stimuli at contrast detection threshold in observers with normal vision. These were obtained by searching PubMed using the term “binocular summation” (401 hits on January 19, 2018) and then screening each study to determine its methodological details, yielding 52 studies. A further 13 relevant studies were included that were identified through secondary searches and the authors’ knowledge of the literature, giving a total of 65 studies (see Appendix A for a full PRISMA flow diagram). In some cases summation data were given in tables or in the text; in others they were estimated from published figures using computer software. In instances where data for a control and a clinical group were reported, we included only the control data.

We performed the meta-analysis using estimates of summation ratios expressed in decibel (dB) units, defined as 20 × log10(BSR), where BSR is the ratio of monocular to binocular thresholds expressed in Michelson contrast (or equivalently the ratio of binocular to monocular contrast sensitivity). In these units, a summation ratio of 2 is equivalent to 6 dB, a ratio of 1 is equivalent to 0 dB, and a ratio of √2 is equivalent to 3 dB. Where possible, we calculated the mean for each observer across all experimental conditions (e.g., different spatial or temporal frequencies, depending on the study) and then computed a mean and standard deviation across observers, and used this to estimate 95% confidence intervals using the approximation ±1.96 × *SE*. In other studies, data for individual observers were not available, and we estimated the standard deviation by pooling variances across conditions assuming negligible covariance between conditions (which is implausible, but gives an upper bound on the variance estimate). Where standard deviations (or standard errors) were given in linear units, we converted these to dB units before averaging. For some studies it was only possible to obtain the mean, and so a measure of variance is not given. The full meta-analysis summary table is included in Appendix A.

### Meta-Analysis: Results

**Figure 1 fig1:**
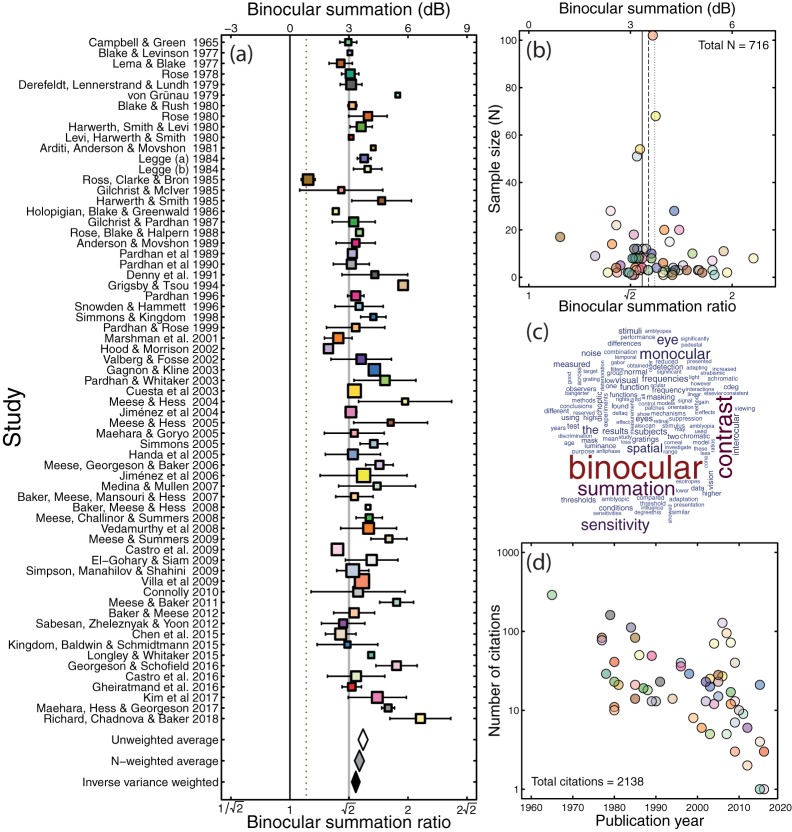
Meta-analysis summary. (a) Forest plot of binocular summation across 65 studies. Square symbol width is proportional to the log of the sample size plus one. Error bars give 95% confidence intervals, estimated using the approximation ±1.96 × *SE*. The black vertical line gives the line of no effect, where binocular and monocular sensitivities are equal. The dashed vertical line gives an estimate of probability summation for two independently noisy signals. The gray vertical line gives the traditional value of √2. The white diamond gives the average across all studies (3.72 dB, or a ratio of 1.53), weighting each study equally (ignoring sample size). The gray diamond gives the average weighted by the sample size of each study (3.54 dB, or a ratio of 1.50). The black diamond gives the average weighted by the inverse variance of each study (3.35 dB, or a ratio of 1.47). This latter estimate comprises only 55 studies, as a measure of variance was unavailable for 10 studies. The width of the diamonds spans the 95% confidence intervals. (b) Funnel plot showing sample size plotted against binocular summation for all 65 studies. The distribution of summation ratios is approximately symmetrical about the means (with the dotted, dashed, and solid lines corresponding to the white, gray, and black diamonds from Panel a). (c) Word cloud showing the most frequent words used in the abstracts of studies included in the meta-analysis. (d) Number of citations per article included (obtained from Web of Knowledge on January 29, 2018), plotted against year of publication. Articles with no citations are omitted. Colors in Panels b and d correspond to those in Panel a.

Figure 1a shows a summary of the meta-analysis results represented as a forest plot. Each row denotes a separate study, with the horizontal placement of the symbol giving the mean level of binocular summation for that study, and error bars giving the 95% confidence intervals. For the vast majority of studies (all but 2), the lower bound of the confidence intervals exceeded a summation ratio of 1 (no summation, given by the solid black vertical line) and 1.1 (dashed brown line)—a level that has come to be associated with probabilistic summation of two independent noisy inputs (Meese & Summers, 2012; Tyler & Chen, 2000).[Fig-anchor fig1]

Much less clear from inspecting the individual means is whether the population of studies shows summation above the classical value of √2. To determine this, we averaged across studies to produce aggregate estimates of summation. When each study is given equal weight (regardless of sample size), the mean level of summation was 1.53, as shown by the white diamond at the foot of Figure 1a. The lower bound of the 95% confidence interval was comfortably above the √2 level. We also calculated a weighted average, where each study was multiplied by its sample size, and the total divided by the sum of the weights (gray diamond in Figure 1a). This slightly reduced the mean summation ratio (to 1.50), but left the lower bound of the confidence interval above √2 (at 1.46). Finally, we weighted studies by the inverse of the variance across participants (black diamond in Figure 1a). An estimate of variance was available for 55 studies, with five of the remaining studies featuring only one participant, and the remaining five failing to report a usable measure of variability. Across these 55 studies, the weighted mean was 1.47, with the lower bound of the 95% confidence interval at 1.43. Therefore all three methods for weighting the summation ratios produced an average value that was significantly above the classical estimate of √2.

**Figure 2 fig2:**
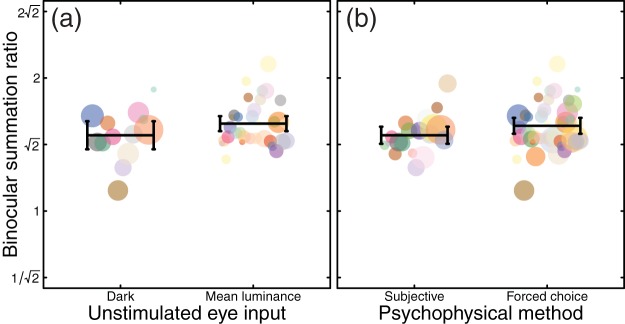
Effect of methodology on binocular summation. (a) Compares studies in which the unstimulated eye (in monocular conditions) viewed mean luminance, with studies in which it wore a patch and was therefore dark. (b) Compares studies that used criterion free forced-choice methods with studies that used other methods (such as the method of adjustment, or yes/no tasks). In both panels, data from a single study have a color consistent with Figure 1a, and symbol diameter is proportional to the base-10 logarithm of sample size (plus an added constant to avoid sizes of zero for studies with only one participant). Black horizontal lines in give the unweighted means across studies, and error bars give 95% confidence intervals.

We next asked which methodological factors might lead to the interstudy variability in summation ratios. One methodological difference between studies is the way in which the unstimulated eye is treated during monocular conditions. In many studies (particularly older studies and those with a clinical focus) the unstimulated eye wore a patch, and was therefore completely dark during monocular conditions (*N* = 13). Other studies use specialist equipment, such as stereoscopes, virtual reality headsets, or stereo shutter goggles to present mean luminance to the unstimulated eye on monocular trials (*N* = 33). In these studies, trials from different conditions (binocular vs. monocular presentation) can be interleaved so that the participant is unaware of whether one or both eyes are being stimulated on a given trial. It has been suggested that luminance from an otherwise unstimulated eye can have an effect on sensitivity to periodic stimuli presented to the other eye (Denny, Frumkes, Barris, & Eysteinsson, 1991; Yang & Stevenson, 1999), and this dichoptic “zero frequency” masking might be expected to influence binocular summation. Studies in which the unstimulated eye saw mean luminance on monocular trials reported slightly greater levels of binocular summation than studies involving patching (mean ratios of 1.57 vs. 1.48; Figure 2a). However, a Welch’s *t* test comparing summation ratios from studies using these two methodologies (12 studies in which the method was not clearly stated, and 7 studies using a translucent occluder were omitted) found that the difference was not significant (*t* = 1.43, *df* = 19.25, *p* = .17).[Fig-anchor fig2]

A second difference across studies concerns the psychophysical methodology used to estimate thresholds. Many older studies used techniques such as the method of adjustment or yes/no tasks to estimate thresholds (*N* = 19). These methods are subject to bias, from participants adjusting their criteria for setting thresholds (or for responding yes or no), which might be more severe in studies where the condition being tested (monocular or binocular) was made explicit by the use of a patch. More recent work (*N* = 46) has tended to use bias-free forced-choice methods to avoid such problems. Bias-free methods produced slightly greater levels of summation (mean ratio 1.56) compared with other methods (mean ratio 1.48; see Figure 2b). Nevertheless, a Welch’s *t* test found no significant difference between these two methodologies (*t* = 1.70, *df* = 40.96, *p* = .10).

## Summation Varies With Spatiotemporal Stimulus Properties

**Figure 3 fig3:**
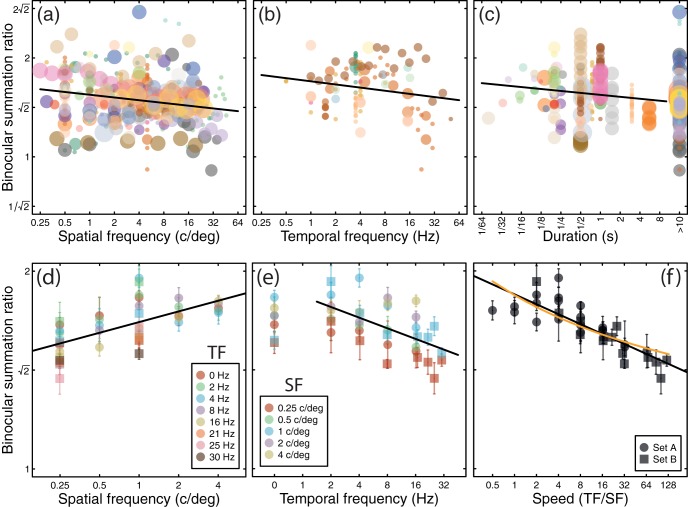
Effects of spatial and temporal stimulus properties on binocular summation. The upper row shows data from the meta-analysis, plotting summation as a function of spatial frequency (a), temporal frequency (b), and stimulus duration (c) using the same symbol size and color conventions as in Figure 2. In (c), studies that allowed unlimited inspection time are assigned a duration of >10 s. The lower row shows the results of two experiments measuring binocular summation as a function of spatial frequency (d), temporal frequency (e), and speed (f), given by the ratio of temporal frequency to spatial frequency, in deg/s. The same data are reproduced in each panel, except that the 0 Hz data are omitted from Panel f. Error bars indicate ±1 *SE* of the mean across observers (*N* = 4 for each data point). Black lines in all panels are best fitting regression lines (on log-transformed values), and the orange curve in (f) is the prediction of Equation 2 when the exponent *m* depends on stimulus speed (see text for details).

A further source of methodological variability across studies concerns the spatiotemporal properties of the stimuli used. To explore these factors, we reanalyzed the meta-analysis studies to average across all observers from a given study that had completed a specific spatiotemporal condition. We summarize the results in three ways in Figure 3: as a function of spatial frequency (Figure 3a), temporal frequency (Figure 3b) and presentation duration (Figure 3c). Linear regression (on logarithmic values) showed significant negative effects of spatial frequency (*t* = −4.00, *p* < .001) and temporal (flicker) frequency (*t* = −2.06, *p* < .05) but not duration (*t* = −1.68, *p* = .09). However, in principle these effects could stem from methodological or sampling differences across studies. To ensure that the effects of spatiotemporal frequency are robust, we would ideally seek to replicate them within a single study.[Fig-anchor fig3]

Previous studies have manipulated spatial (Campbell & Green, 1965; Ross, Clarke, & Bron, 1985; Simpson, Manahilov, & Shahani, 2009) and/or temporal (Baker & Meese, 2012; Rose, 1980) frequency experimentally, sometimes finding systematic effects on binocular summation. Yet we found no published study reporting summation as a function of both spatial and temporal frequency that manipulated both variables across a wide range. Such a study is necessary to validate the findings from the meta-analysis while controlling for potential methodological confounds (e.g., if spatiotemporal frequency covaried with stimulus size, psychophysical task, equipment used, or other factors such as mean luminance). Fortunately, archival data were available that met these requirements. Two experiments testing a wide range of different spatiotemporal conditions (termed Set A and Set B), were conducted at Aston University during 2004 and 2005. These data have previously been reported only in abstract form (Georgeson & Meese, 2005, 2007), but are presented here in full for the first time. Methodological details are available in Appendix B.

### Spatiotemporal Study: Results

Binocular summation was apparent in all conditions tested with both stimulus sets, with an overall average summation ratio of 1.65 (4.33 dB). We plot the results in three ways in Figure 3d–3f. Plotting binocular summation as a function of spatial frequency (collapsing across all temporal conditions) reveals an increase in summation with increasing spatial frequency (Figure 3d). The best fit regression line (in logarithmic units) had a highly significant positive slope of 0.05 (*R*^2^ = 0.40, *t* = 5.25, *p* < .001), meaning that an increase in spatial frequency of a factor of 10 will increase summation by around 12% (1 dB). This effect is in the opposite direction to the effect of spatial frequency across the studies in the meta-analysis (see Figure 3a), which showed a slight negative effect of spatial frequency. We discuss possible explanations for this discrepancy in the next section.

There was a significant negative effect of temporal frequency (*R*^2^ = 0.33, *t* = −4.14, *p* < .001) with a slope of −0.05 (excluding the static conditions which had a nominal frequency of 0 Hz). This suggests that a tenfold increase in temporal frequency will reduce summation by around 12% (see Figure 3e), broadly consistent with the estimate from the meta-analysis (a slope of −0.03; Figure 3b).

Since summation increases with spatial frequency and decreases with temporal frequency, the data are consistent with an effect of implied stimulus speed. This measure, defined as the ratio of temporal to spatial frequency in deg/s, is a scalar quantity that has no implied direction. Replotted as a function of speed (Figure 3f), binocular summation shows a remarkably lawful decline, as indexed by the highly significant linear regression (*R*^2^ = 0.70, *t* = −8.7, *p* < .001) with a slope of −0.05 in logarithmic units (black line). This holds across speeds varying over more than two orders of magnitude in the present experiment. Because summation depends on the strength of the exponent in Equation 2, it follows that this exponent (*m*) can be considered a function of stimulus speed. Specifically, the function *m* = 1.14 + 0.28 × log_10_(TF/SF), where TF is temporal frequency (in Hz) and SF is spatial frequency (in c/deg), provides the best least squares fit to the data, as shown by the orange curve in Figure 3f. In short, increasing the early nonlinearity (*m*) with speed could account for the observed decrease in binocular summation.

### Spatiotemporal Study: Discussion

The effect of temporal frequency on binocular summation is consistent between the meta-analysis and the experiment reported here. However, higher spatial frequencies were associated with weaker summation in the meta-analysis, but stronger summation in the stand-alone experiment. What might account for this puzzling discrepancy?

One key factor that can act to depress empirical summation ratios is the sensitivity difference between the two eyes. In many studies, summation ratios are calculated by comparing binocular sensitivity with that of the more sensitive eye. When the two eyes are approximately equal this should give an accurate estimate of summation. However, as the sensitivity difference between the eyes increases, the “boost” from the less sensitive eye becomes weaker. At low spatial frequencies, sensitivities are usually well balanced, but at higher frequencies optical and neural factors penalize the weaker eye and reduce its sensitivity (e.g., Pardhan, 1996). Therefore in the studies included in the meta-analysis, the apparent spatial frequency effect might in fact be due to monocular asymmetries in sensitivity. We next explore how differences in monocular sensitivity can influence estimates of binocular summation.

## Individual Differences in Interocular Sensitivity Predict Summation

Even individuals with intact binocular vision often exhibit asymmetries in sensitivity across the two eyes. For example, Pardhan (1996) measured binocular summation in older and younger participants at both 1 and 6 c/deg. In the older group, interocular sensitivity ratios (worse eye/better eye) showed a greater imbalance at the higher spatial frequency (mean ratio 0.74) than at the lower frequency (mean ratio 0.85), and on a scatterplot of individual data points there was a strong relationship between the interocular sensitivity ratio and binocular summation. Such asymmetries will influence the levels of binocular summation measured experimentally, depending on precisely how summation is calculated.

By plotting summation for observers with naturally varying amounts of interocular sensitivity difference, we can measure the change in summation that occurs in individuals with large asymmetries, and also estimate the true level of neural binocular summation. We first do this by replotting data from a subset of 21 studies from the meta-analysis for which individual monocular thresholds for both eyes were available (total *N* = 239). However, because the diversity of stimulus conditions used across studies could influence the results (e.g., via the effects of spatiotemporal frequency reported above), we replicate our findings by collecting new data in a group of 41 observers using common stimulus conditions. Methodological details for this experiment, which was conducted at the University of York during 2017, are available in Appendix C.

### Results of Individual Differences Analysis

**Figure 4 fig4:**
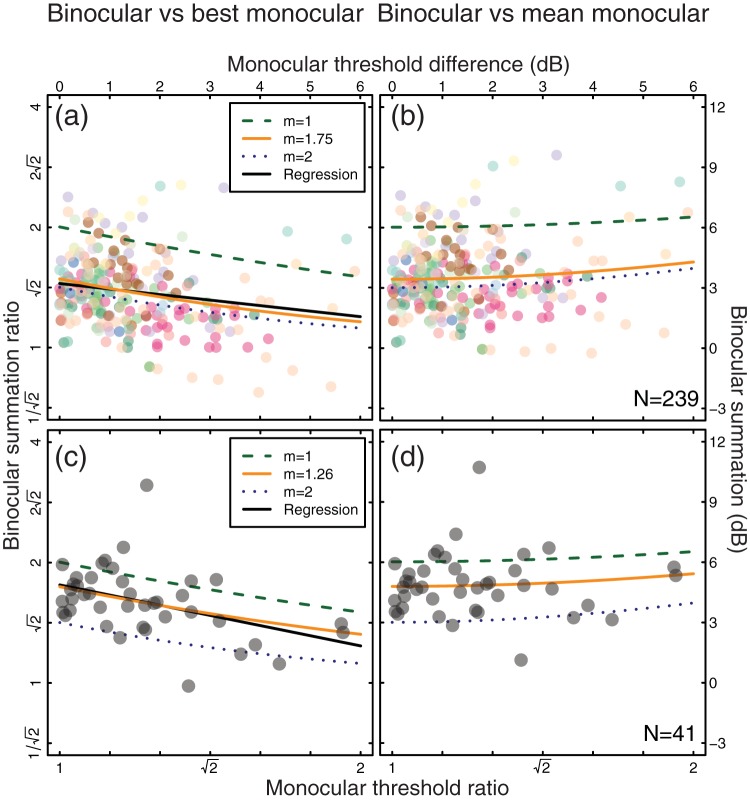
Change in binocular summation as a function of monocular sensitivity imbalance. (a, c) Summation is calculated as the ratio of the binocular threshold and better of the two monocular thresholds. (b, d) Summation is calculated as the ratio of the binocular threshold and the average of the two monocular thresholds. In all panels, a monocular threshold ratio of 1 indicates equal monocular sensitivities, and a ratio of 2 means that one eye was twice as sensitive as the other. Each data point represents one observer, either from studies in the meta-analysis with diverse spatiotemporal conditions (Panels a and b; *N* = 239), or from a stand-alone experiment with constant stimulus properties (Panels c and d; *N* = 41). The black curves in Panels a and c are the best fitting regression line (using logarithmic values), with slopes of −0.3 (a) and −0.5 (c) and *y* intercepts of 3.20 dB (a) and 4.89 dB (c). The remaining curves show summation predictions for a linear transducer (green dashed curves), square law transducer (blue dotted curves), and best fitting exponents (orange solid curves) under both calculation schemes.

Binocular summation is plotted as a function of the threshold difference between the eyes in Figure 4. In the upper row the data are from a subset of 21 studies from the main meta-analysis, and in the lower row the data are from a single experiment. The monocular threshold difference was calculated by taking the absolute difference between left and right eye thresholds (in dB units). Binocular summation was calculated in two ways: first, by subtracting the binocular threshold from the lower of the two monocular thresholds (in dB units, plotted in Figure 4a and 4c), and second, by subtracting the binocular threshold from the average of the two monocular thresholds (plotted in Figure 4b and 4d).[Fig-anchor fig4]

When summation is calculated relative to the best monocular threshold, there is a clear downward trend in both data sets, summarized by the significant negative correlations (Figure 4a, *R* = −0.19, *p* < .01; Figure 4c, *R* = −0.43, *p* < .01) and best fit linear regressions (black curves) with slopes of −0.28 (Figure 4a) and −0.5 (Figure 4c). The regression intercepts were 3.20 dB (Figure 4a) and 4.89 dB (Figure 4c). These intercepts imply binocular summation ratios of 1.45 (Figure 4a) and 1.76 (Figure 4c) when the eyes are equally sensitive. The slope of −0.5 (or −0.3) means that for every 1 dB difference in sensitivity between the eyes, the measured level of binocular summation reduces by 0.5 dB (or 0.3 dB).

This trend is qualitatively consistent with the predictions of both linear (green dashed curve) and quadratic (blue dotted curve) summation models, determined by penalizing the contribution of one eye in Equation 2 by varying amounts, for exponents *m* = 1 and *m* = 2. Permitting the exponent to vary resulted in best-fitting estimates of *m* = 1.75 (Figure 4a) and *m* = 1.26 (Figure 4c), given by the orange solid curves. In the absence of an interocular sensitivity difference, this implies summation ratios of 3.44 dB (a ratio of 1.49, Figure 4a) and 4.80 dB (a ratio of 1.74, Figure 4c), significantly higher (Figure 4a, *t* = 4.88, *df* = 238, *p* < .001; Figure 4c, *t* = 2.78, *df* = 40, *p* < .01) than the group averages of 2.76 dB (a factor of 1.37, Figure 4a) and 4.06 dB (a factor of 1.6, Figure 4c). These results show how even relatively modest monocular sensitivity differences can influence population estimates of summation when it is calculated relative to the best monocular threshold (as is typical in many studies).

Figure 4b and 4d replot the same data, but this time binocular summation was calculated relative to the average of the two monocular thresholds. Under this scheme, summation ratios are predicted to increase very slightly for larger monocular imbalances, because the higher monocular threshold in the weaker eye elevates the mean monocular threshold. This is borne out by the very slight positive trend in the data points across both panels. Calculated in this way, the group average summation ratios were 3.56 dB (a factor of 1.51, Figure 4b) and 4.88 dB (a factor of 1.75, Figure 4d). The curves in Figure 4b and 4d show simulated summation levels for different transducer exponents as a function of interocular asymmetry (implemented in the model by attenuating the input contrast to the weaker eye). For a linear transducer (*m* = 1, green dashed curve), a monocular difference of 6 dB (a factor of 2) increases empirical summation by 0.5 dB (around 6%). For a square law transducer (*m* = 2, blue dotted curve), the expected increase is 1 dB (12%). Again, the replotted data follow this trend qualitatively, and are well-described by the orange curve with exponents *m* = 1.75 (Figure 4b) and *m* = 1.26 (Figure 4d) that was fit to the data in Figure 4a and 4c.

### Individual Differences Analysis: Summary

By replotting a subset of the meta-analysis data and confirming with a new experiment, we demonstrated that individual differences in monocular sensitivity can affect empirically measured binocular summation. Overall, the data are consistent with monocular exponents of *m* = 1.75 (a true binocular summation ratio of around 1.49) across studies with varying spatiotemporal properties (Figure 4a and 4c), and *m* = 1.26 (a true binocular summation ratio of around 1.74) when methodological details are held constant (Figure 4b and 4d).

## General Discussion

We revisited the extent to which contrast sensitivity improves for two eyes compared with one. Across a meta-analysis of 65 studies, and two additional experiments, we demonstrated conclusively that binocular summation is significantly greater than the traditional value of √2, and considered several factors that can affect empirical estimates of this parameter. Spatiotemporal frequency, and the sensitivity difference between the eyes both have an influence on empirical summation estimates. These effects suggest that there is no single canonical level of summation (as was originally proposed by Campbell & Green, 1965), but instead a range of values between approximately √2 and 2, depending on precise experimental conditions. We now discuss several of these factors in greater detail, and consider their importance for the clinical assessment of binocular function, and best practice for future studies.

### Do Higher Spatial Frequencies Increase or Decrease Summation?

As demonstrated in Figure 4a and 4c, imbalances in monocular sensitivity can have a negative impact on binocular summation when it is calculated relative to the best monocular threshold. Since this is standard practice for many published studies (e.g., Chen et al., 2015; Longley & Whitaker, 2016; Pardhan & Rose, 1999), monocular asymmetries at higher spatial frequencies are a plausible explanation for the apparent changes in binocular summation shown in Figure 3a. But in the spatiotemporal experiment, the raw monocular data were pooled to calculate a single threshold. Might this have led to spurious increases in summation at high spatial frequencies, as illustrated in Figure 4b and 4d? This is unlikely for two reasons. First, the effects are rather modest, even for quite large sensitivity differences (i.e., <1 dB for a 6 dB threshold difference). Second, monocular sensitivity differences of that magnitude would reduce the slope of the psychometric function used for estimating the pooled monocular threshold (because the pooled data would come from two underlying psychometric functions with a relative lateral displacement). The (geometric) mean slopes were almost identical across the binocular (mean Weibull β = 2.384) and monocular (mean Weibull β = 2.377) conditions, and showed no significant differences (*p* > .05). We therefore conclude that the increase in summation at higher spatial frequencies is a genuine effect, but one that was previously obscured in published studies by methodological factors.

### Binocular Summation in Clinical Populations

Several studies appear to show that binocular summation is negligible in amblyopia (Harwerth, Smith, & Levi, 1980; Lema & Blake, 1977; Pardhan & Gilchrist, 1992), and from this it was often concluded that neural binocular mechanisms were absent or compromised in this condition. However, one of the key symptoms of amblyopia is a reduction of contrast sensitivity in the amblyopic eye, particularly at higher spatial frequencies (Hess & Howell, 1977). The apparent absence of binocular summation could be due to an extreme version of the effect shown in Figure 4a and 4c, whereby monocular imbalances reduce empirical summation estimates (to negligible levels). By adjusting the monocular contrasts to compensate for the sensitivity difference (a technique originally developed for estimating binaural summation; Shaw, Newman, & Hirsh, 1947), normal levels of binocular summation become apparent in individuals with amblyopia (Baker, Meese, Mansouri, & Hess, 2007), indicating that neural binocular mechanisms remain intact. A similar apparent loss of summation can be induced in observers with normal binocular vision by reducing the luminance to one eye using a neutral density filter (Baker et al., 2007; Gilchrist & McIver, 1985; Richard, Chadnova, & Baker, 2018). This reduces sensitivity without affecting contrast, and can be similarly compensated by boosting the contrast in the filtered eye (Baker et al., 2007). Future clinical studies must therefore exercise methodological diligence in using binocular summation to assess binocular function, especially in situations where monocular sensitivities may be unequal.

### What Is the Best Way to Measure Summation?

Our results here point to some guidelines for how best to estimate neural binocular summation in future studies. Patching of the unstimulated eye should be avoided if at all possible, ideally by using equipment (stereoscopes, shutter goggles or virtual reality hardware) designed for binocular presentation. If this is not possible, then placing a frosted occluder in front of the unstimulated eye will ensure that it views an uncontoured field of nearly the same mean luminance. Unbiased forced-choice methods using adaptive staircases (or similar) are preferable to techniques in which the participant adjusts the stimulus contrast to reach some internal criterion (as this is subject to bias), or eye-chart-based methods (for which the set of possible thresholds is typically quantized to the range of stimuli on the chart).

Monocular thresholds should always be measured for each eye. If there are substantial differences in sensitivity across the eyes, then one option is to use a procedure in which the components of the binocular stimulus are normalized to the monocular detection thresholds (Baker et al., 2007). If this is not possible, then modeling the sensitivity difference can provide unbiased estimates of summation by calculating an attenuation weight for the weaker eye, finding the best exponent to describe the amount of summation measured, and inferring the level of summation that would be expected if sensitivities were equal (e.g., Figure 4a and 4c). For moderate sensitivity differences (e.g., <3 dB), averaging the monocular thresholds is preferable to using the threshold of the better eye to calculate summation ratios, though this can slightly overestimate binocular summation (see Figure 4b and 4d).

### Appropriate Sample Sizes for Estimating Binocular Summation

The inverse variance weighted aggregate measure of binocular summation (given by the black diamond in Figure 1a) implies an effect size (Cohen’s *d*) of around 31 for detecting the existence of binocular summation (i.e., relative to a summation ratio of 1). This unusually large effect size means that even a study with only two participants should be capable of detecting the presence or absence of binocular summation (using a one-sample *t* test) with 99.99% power. When comparing binocular summation to the canonical value of √2, the effect size is still very large (*d* = 3.22), meaning that a study with three participants has over 95% power. Our meta-analysis therefore demonstrates that the tradition of small sample sizes in psychophysical studies is often appropriate, given the magnitude and stability of the effects involved, and the precision of the measurement techniques.

### Summation for Other Visual Cues

The present study was confined to the investigation of binocular summation of contrast at threshold using psychophysical techniques. Many of the studies we encountered while conducting the meta-analysis reported binocular summation for other visual tasks, including binocular summation for visual acuity, the detection of luminance at absolute threshold, and electroencephalographic measures of binocular function. Understanding how the visual system integrates different cues across the eyes, and how the findings for contrast apply to different domains, will require further study. However, we note that the same general framework for signal combination and suppression that we discuss here and in our other work (Georgeson, Wallis, Meese, & Baker, 2016; Meese et al., 2006) has been successfully applied to understand binocular combination of cues such as motion (Maehara, Hess, & Georgeson, 2017) and contrast modulation (Georgeson & Schofield, 2016), as well as summation across space (Meese & Summers, 2007), time, and orientation (Meese & Baker, 2013), and also to make accurate predictions regarding neural responses (Baker & Wade, 2017).

### Conclusions

We asked whether binocular summation was greater than the widely cited value of √2. A meta-analysis of 65 studies involving 716 observers showed that summation is significantly above this level, and furthermore that it was influenced by the spatial and temporal properties of the visual stimulus. We then showed empirically that stimulus speed (the ratio of temporal to spatial frequency) determines summation in a systematic way, such that low speeds produce greater summation than high speeds. Finally, we found that the difference in monocular sensitivities can affect empirical estimates of summation. Overall, estimates of binocular summation fall within the range between √2 and 2, depending on stimulus properties, and this range of values reflects speed-related changes in the strength of an early nonlinearity occurring prior to binocular combination.

## Figures and Tables

**Table A1 tbl1:** Meta-Analysis Summary Table

Study	*N*	BSR (dB)	*SD* (dB)	Citations	Method	Setup
Campbell and Green (1965)	2	2.966	.310	289	MOA	Occluder
Blake and Levinson (1977)	1	3.046		77	MOA	Stereoscope
Lema and Blake (1977)	4	2.578	.603	83	MOA	Occluder
Rose (1978)	8	3.063	.630	29	MOA	Occluder
Derefeldt, Lennerstrand, and Lundh (1979)	12	3.110	.976	161	MOA	Patch
Von Grünau (1979)	1	5.480		0	2AFC	Patch
Blake and Rush (1980)	3	3.174	.199	10	MOA & 2AFC	Stereoscope
Rose (1980)	6	3.964	1.222	11	MOA	Occluder
Harwerth, Smith, and Levi (1980)	8	3.620	.813	23	RT	Patch/diffuser
Levi, Harwerth, and Smith (1980)	1	3.111		41	2AFC	Stereoscope
Arditi, Anderson, and Movshon (1981)	1	4.235		21	2AFC	Stereoscope
Legge (1984a)	4	3.773	.356	112	2AFC	Stereoscope
Legge (1984b)	2	3.950	.520	112	2AFC	Stereoscope
Ross, Clarke, and Bron (1985)	17	.926	.745	83	2AFC	Patch
Gilchrist and McIver (1985)	2	2.608	1.524	21	MOA	Not stated
Harwerth and Smith (1985)	3	4.660	1.340	14	MDL	Not stated
Holopigian, Blake, and Greenwald (1986)	2	2.330	.100	50	2AFC	Stereoscope
Gilchrist and Pardhan (1987)	8	3.242	1.575	19	2AFC	Not stated
Rose, Blake, and Halpern (1988)	3	3.532	.093	18	2AFC	Stereoscope
Anderson and Movshon (1989)	4	3.342	1.015	49	MOA	Stereoscope
Pardhan, Gilchrist, and Douthwaite (1989)	8	3.170	.140	13	2AFC	Stereoscope
Pardhan, Gilchrist, Douthwaite, and Yap (1990)	8	3.120	1.320	13	2AFC	Stereoscope
Denny, Frumkes, Barris, and Eysteinsson (1991)	3	4.313	1.475	23	2AFC	Stereoscope
Grigsby and Tsou (1994)	11	5.750		14	Yes/No	Translucent patch
Pardhan (1996)	8	3.346	.602	36	2AFC	Patch
Snowden and Hammett (1996)	3	3.514	1.084	40	2AFC	Goggles
Simmons and Kingdom (1998)	2	4.240	.463	29	2AFC	Goggles
Pardhan and Rose (1999)	4	3.340	1.511	8	2AFC	Stereoscope
Marshman, Dawson, Neveu, Morgan, and Sloper (2001)	14	2.457	1.338	6	2AFC	Occluder
Hood and Morrison (2002)	9	1.957		13	MAL	Occluder
Valberg and Fosse (2002)	10	3.620	2.480	23	Yes/No	Not stated
Gagnon and Kline (2003)	28	4.292		20	3AFC	Patch
Pardhan and Whitaker (2003)	10	4.830	2.540	5	2AFC	Occluder
Cuesta, Anera, Jiménez, and Salas (2003)	54	3.280		25	3AFC	Not stated
Meese and Hess (2004)	2	5.853	1.707	70	2AFC	Stereoscope
Jiménez and Anera (2004)	18	3.100	.650	12	3AFC	Not stated
Meese and Hess (2005)	2	5.120	1.354	28	2AFC	Stereoscope
Maehara and Goryo (2005)	3	3.270	1.345	23	2AFC	Stereoscope
Simmons (2005)	4	4.262	.709	23	2AFC	Stereoscope
Handa, Shimizu, Mukuno, Kawamorita, and Uozato (2005)	12	3.200	2.480	15	Eyechart	Not stated
Meese, Georgeson, and Baker (2006)	5	4.550	.795	128	2AFC	Goggles
Jiménez, Villa, Anera, Gutiérrez, and del Barco (2006)	68	3.743	9.291	27	Not stated	Not stated
Medina and Mullen (2007)	3	4.429	1.726	5	2AFC	Translucent patch
Baker, Meese, Mansouri, and Hess (2007)	3	3.279	.839	95	2AFC	Goggles
Baker, Meese, and Hess (2008)	1	3.970		72	2AFC	Goggles
Meese, Challinor, and Summers (2008)	3	4.027	.588	17	2AFC	Goggles
Vedamurthy, Suttle, Alexander, and Asper (2008)	20	3.998	3.239	12	2AFC	Goggles
Meese and Summers (2009)	3	5.030	.808	40	2AFC	Goggles
Castro, Jiménez, Hita, and Ortiz (2009)	28	2.415	.517	13	Not stated	Not stated
El-Gohary and Siam (2009)	15	4.155	2.640	0	Eyechart	Not stated
Simpson, Manahilov, and Shahani (2009)	51	3.192	2.969	7	2AFC	Occluder
Villa, Jiménez, Anera, Gutiérrez, and Hita (2009)	102	3.660		3	Not stated	Occluder
Connolly (2010)	12	3.464	4.222	10	Yes/No	Opaque occluder
Meese and Baker (2011)	3	5.422	.762	9	2AFC	Goggles
Baker and Meese (2012)	9	3.264	1.592	2	2AFC	Goggles
Sabesan, Zheleznyak, and Yoon (2012)	5	2.704	1.260	6	2AFC	Stereoscope
Chen et al. (2015)	22	2.582	1.853	4	2AFC	Patch
Kingdom, Baldwin, and Schmidtmann (2015)	2	2.923	1.112	21	2AFC	Stereoscope
Longley and Whitaker (2016)	2	4.130	.110	1	2AFC	Occluder (black)
Georgeson and Schofield (2016)	7	5.417	1.416	1	2AFC	Goggles
Castro, Soler, Ortiz, Jiménez, and Anera (2016)	12	3.364	2.573	0	4AFC	Not stated
Gheiratmand, Cherniawsky, and Mullen (2016)	4	3.143	.500	3	2AFC	Stereoscope
Kim, Reynaud, Hess, and Mullen (2018)	20	4.440	3.370	0	2AFC	Patch
Maehara, Hess, and Georgeson (2017)	3	4.990	.290	0	2AFC	Stereoscope
Richard, Chadnova, and Baker (2018)	8	6.630	2.231	0	2AFC	Goggles
*Note.* BSR = binocular summation ratio; MOA = method of adjustment; 2AFC = two-interval forced-choice; MDL = method of descending limits; MAL = method of ascending limits; RT = reaction time.						

**Figure A1 fig5:**
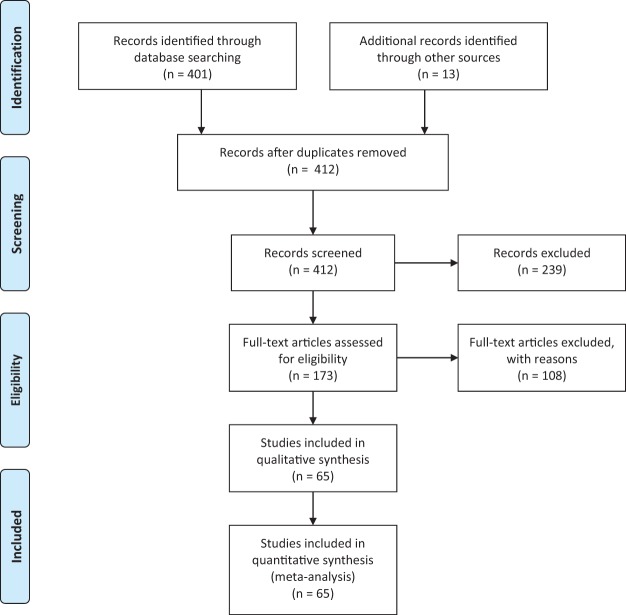
PRISMA flow diagram.

## A Meta-Analysis Summary Table and PRISMA Diagram

[Table-anchor tbl1][Fig-anchor fig5]

## B Spatiotemporal Experiment: Method

Target stimuli were horizontal sine-wave gratings with spatial frequencies of 0.25, 0.5, 1, 2, and 4 c/deg (Set A), or 0.25 and 1 c/deg (Set B). Stimulus contrast was spatially windowed by a circular aperture with smoothed edges and a diameter at half-height of 4°. Stimulus contrast was temporally windowed by one cycle of a raised cosine (duration 500 ms) and within that envelope contrast flickered sinusoidally in counterphase at frequencies of 2, 4, 8, and 16 Hz (Set A) or 2, 4, 8, 16, 21, 25, and 30 Hz (Set B). There was also a static condition, in which the stimulus did not flicker (i.e., a nominal frequency of 0 Hz). All factorial combinations of spatial and temporal frequencies within a set were tested. The experiments were completed by independent groups of naïve observers for Set A (*N* = 4) and Set B (*N* = 4). All observers had no reported history of binocular abnormalities, and wore their prescribed optical correction if required.

Stimuli were generated using a Bits++ video interface (Cambridge Research Systems, Kent, UK) controlled by an Apple Macintosh computer running Matlab, and presented with 14-bit luminance resolution on a gamma-corrected Clinton Monoray monitor running at 150 Hz. The display was viewed through ferro-electric FE1 stereo shutter goggles synchronized with the monitor refresh rate to permit independent control of images to the left and right eyes via frame interleaving. Mean luminance, as seen through the alternating goggles, was 26 cd/m^2^. The goggles ensured that during monocular conditions, the unstimulated eye viewed mean luminance.

Stimuli were presented for 500 ms in one of two intervals, each marked by a beep, with a gap of 500 ms between intervals. In one interval the target was presented, with its contrast determined by a 3-down 1-up staircase algorithm. The other interval was blank. Any given trial could either be monocular (left or right eye) or binocular, and the observer was not informed of this. Stimuli were blocked by spatiotemporal condition. Observers indicated which interval contained the target using a keypad and received auditory feedback regarding accuracy. Staircase algorithms terminated after 100 trials, and each observer repeated the experiment four times, resulting in 20,000 (Set A) or 12,800 (Set B) trials per observer (in Set B there were additional trials in which the stimuli were in antiphase across the eyes, but these data are not reported here). Thresholds for individual observers were estimated by fitting Weibull functions to the psychometric data pooled across all repetitions, and taking the contrast at the 81.6% correct point. Binocular summation was calculated as the difference (in dB units) between monocular and binocular thresholds. Raw data are available online at https://doi.org/10.6084/m9.figshare.6291458.v1.

## C Individual Differences Experiment: Method

Target stimuli were horizontal sine-wave gratings with a spatial frequency of 1c/deg, windowed by a circular aperture with its edges smoothed by a cosine function to a diameter of 5°. They were generated using a ViSaGe stimulus generator (Cambridge Research Systems, Kent, UK) controlled by a PC running Matlab, and presented with 14-bit luminance resolution on a gamma-corrected Clinton Monoray monitor running at 120 Hz. The display was viewed through ferro-electric stereo shutter goggles synchronized with the monitor refresh rate to permit independent control of images to the left and right eyes via frame interleaving. The goggles ensured that during monocular conditions, the unstimulated eye viewed mean luminance.

Stimuli were presented for 100 ms in one of two intervals, each marked by a beep, with a gap of 400 ms between intervals. In one interval the target was presented, with its contrast determined by a 3-down 1-up staircase algorithm. The other interval was blank. Any given trial could either be monocular (left or right eye) or binocular, and the observer was not made aware of this. Observers indicated which interval contained the target using a mouse and received auditory feedback regarding accuracy. Staircase algorithms terminated after 120 trials or 10 reversals (whichever occurred first). Each observer repeated the experiment three times.

Data were pooled across repetitions and thresholds at 75% correct were estimated for each condition using probit analysis. The experiment was completed by 58 observers, who were mostly psychophysically inexperienced and naïve to the purpose of the experiment. Several observers produced data of poor quality (typical in psychophysical studies with inexperienced observers; Baker & Graf, 2009), involving multiple errors for high contrast stimuli that should obviously have been visible. To improve data quality we limited the fitted data to include only target contrasts at and below 0 dB binocularly, and 6 dB monocularly (values above which the stimulus was more than double the average threshold). This produced better fits for many observers. However, we then excluded 17 individuals whose psychometric functions had negative or extremely shallow slopes, implausibly high or low thresholds (exceeding ±3 *SD* of the group mean), or could not be fitted satisfactorily. The final data set consisted of 41 observers whose results met these criteria for all three ocularity conditions. Raw data are available online at https://doi.org/10.6084/m9.figshare.6291458.v1.
